# Insights and innovations to mitigate aviation climate impact by 2030

**DOI:** 10.1038/s44172-024-00290-5

**Published:** 2024-10-21

**Authors:** Kieran Tait, Marius Wedemeyer, Anwar Khan, Mark Lowenberg, Dudley Shallcross

**Affiliations:** 1 Department of Aerospace Engineering (School of Civil, Aerospace and Design Engineering), University of Bristol, Queen’s Building, University Walk, Bristol, BS8 1TR Bristol UK; 2 Department of Aerospace Engineering, Technical University of Munich, Arcisstraße 21, Munich, 80333 Bavaria Germany; 3 Atmospheric Chemistry Research Group, University of Bristol, School of Chemistry, Cantock’s Close, University Walk, Bristol, BS8 1TS UK

**Keywords:** Climate-change mitigation, Aerospace engineering

## Abstract

The aviation sector needs to work fast to address its impact on the environment. A recent small conference in Bristol brought together technologists, climate scientists, policy makers and activists to examine the issues. Here we report on presentations and discussions from the conference, exploring insights, innovations and policy implications critical for significant climate impact mitigation within this decisive decade.

## Teaser

The aviation sector needs to work fast to address its impact on the environment. A small conference in Bristol brought together technologists, climate scientists, policy makers and activists to examine the issues. Here we report on presentations and discussions from the conference, exploring insights, innovations and policy implications critical for significant climate impact mitigation within this decisive decade.

## Introduction

Recent climate predictions state that the critical 1.5 °C warming threshold is likely to be breached this decade^1^. Emitting sectors are therefore, in a position of utmost responsibility to enact policy and technology changes which best mitigate climate impact within this timeframe.

Aviation is one of the most difficult-to-abate sectors. This is due to the long lifespan of aircraft and the technological and regulatory barriers that must be overcome to bring about low-emission flight. Aviation industry workers and researchers must therefore open all channels of communication, on how best to effectively mitigate aircraft climate impact, with a focus on what action can be taken in this pivotal decade.

The Aviation Climate Impact Mitigation by 2030 (ACIM2030) conference, held at the University of Bristol on 6 September 2023, was organised to bring together academics, industry experts and other interested individuals, to discuss short-term solutions to rapidly reduce aviation’s impact on the environment. The day consisted of four sessions which captured the primary solution space to the aviation climate impact problem: (1) aviation climate science and modelling, (2) optimising aircraft operations, (3) alternative fuels and propulsion, and (4) technology and policy developments. Over each of the four sessions, experts gave presentations on their respective research areas and concluded with a panel discussion/Q&A. This report aims to summarise the day’s presentations and discussions, focusing on core science, key findings and open issues.

**Fig. 1:**
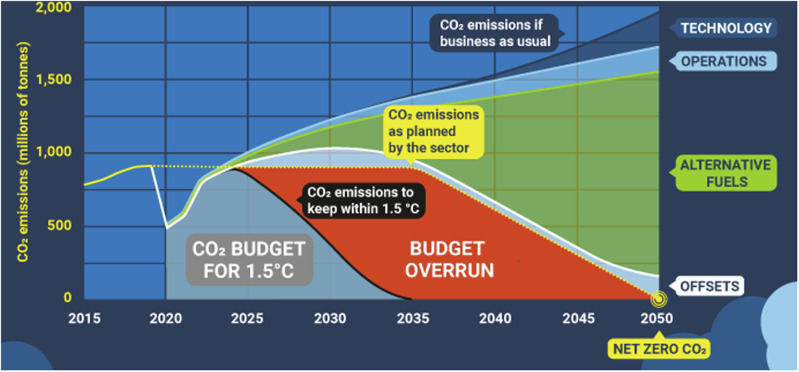
CO_2_ emissions trajectory to 2050. This figure illustrates the projected CO_2_ emissions (in millions of tonnes) over time. It delineates the maximum emission levels permissible within the carbon budget to avoid surpassing the 1.5 °C temperature threshold, emphasizing the necessity to reduce emissions to zero to stay within this limit. Credit: Stay-Grounded^7^. Distributed under a creative commons license https://creativecommons.org/licenses/by/4.0/.

**Fig. 2:**
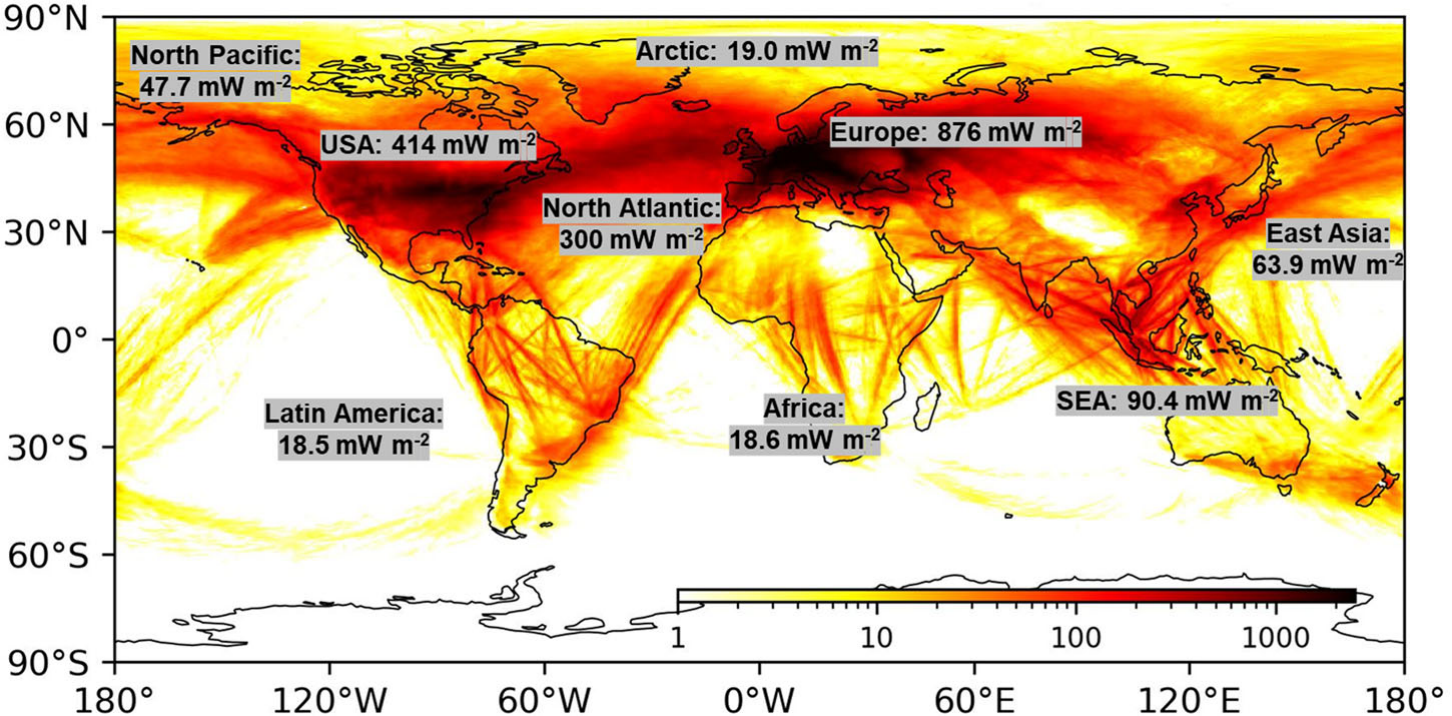
2019 Global annual mean net radiative forcing (RF) of contrail cirrus. This figure displays the global distribution of annual mean net RF due to contrail cirrus for the year 2019. It represents areas where persistent contrails contribute to net RF > 0. Credit: R. Teoh et al.^12^. Distributed under a Creative Commons 4.0 license https://creativecommons.org/licenses/by/4.0/.

**Fig. 3:**
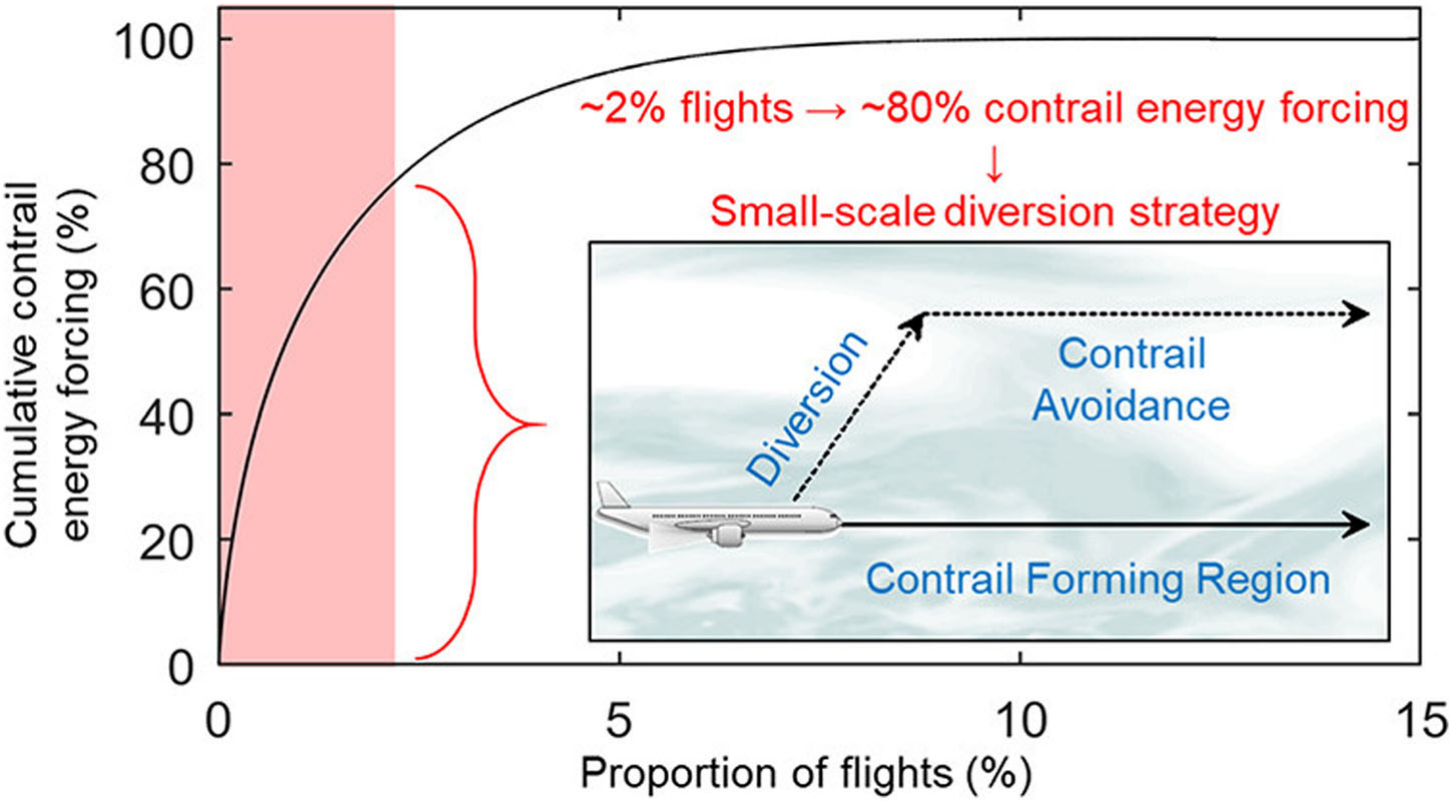
Schematic representation of contrail avoidance strategy. Credit: Reprinted with permission from R. Teoh et al.^29^. Copyright 2024 American Chemical Society.

## Motivation

To set the context for the day and to convey a sense of urgency around the situation, two aviation climate campaigners were invited to present. Both are from prolific non-governmental organisations (NGOs) centred around aviation and the environment. They gave an overview of current climate projections, limitations to current solutions, and alternative outlooks on the road to net-zero aviation.

### Finlay asher (safe landing NGO)

Asher presented an aviation worker’s perspective on the severity of the climate crisis and the need to reduce aviation’s environmental impact this decade. He framed anthropogenic climate change as a budgeting problem, stressing the importance of adhering to carbon budgets consistent with the 1.5 °C pathway. “We have less than a decade before it runs out across all emitting sectors”^2^. Policymakers must, therefore, deliver a sub-budget for aviation, which incentivises drastic action on both national and international aviation emissions and includes the mitigation of non-carbon (non-CO_2_) effects.

The traditional decarbonisation approach focuses on efficiency improvements, carbon offsetting, sustainable aviation fuels (SAFs) and zero-emissions aircraft. Efficiency improvements have stalled in recent years (1−2% per annum), in the face of ever-increasing demand growth (4−5% per annum)^3^. Carbon offsetting is shown to be ineffective, due to a lack of available land on the planet^4^. SAFs and novel aircraft/engine technologies are promising prospects, projected to reduce carbon emissions by  ~80% by 2050. However, there are major issues with feedstock supply and competition with other sectors for the limited resources required to produce SAFs, and it will likely take decades for hydrogen and electric aircraft to enter into service^3^. These solutions are, therefore, inadequate for delivering the necessary trajectory of emissions reductions, to avoid blowing the budget. Instead, deep emissions cuts are required this decade (Fig. 1).

Asher believes this could take the form of non-CO_2_ targeted measures, such as avoidance of condensation trails (contrails), targeted SAF usage and demand reduction policies, such as frequent flyer levies and redistribution of air traffic capacity to developing countries.

### Alethea Warrington (possible NGO)

Warrington spoke about the urgent need for policy change in the aviation industry due to the escalating climate crisis, critiquing the proposed strategy of the JetZero Council for its reliance on external reduction measures like CORSIA^5^ and offsetting, and for “essentially excluding non-CO_2_ emissions”^6^.

Warrington mentioned the Jevons Paradox, illustrating how increased efficiency in aviation paradoxically leads to greater overall fuel consumption^7^. Addressing alternative fuels, Warrington noted, “Every alternative fuel target has been missed, in most cases, by orders of magnitude.”^8^. Furthermore, she expressed scepticism around the feasibility of negative emissions technologies at scale and the effectiveness of carbon offsetting, considering increasing climate risks like forest fires.

Warrington concluded her presentation by asserting that the future of aviation must integrate demand management with short-term technological solutions, advocating for a balanced approach until zero-emissions solutions are fully realised and the fossil-fuel powered fleet is phased out^9^.

## Aviation climate science and modelling

Improving scientific understanding of aviation’s non-CO_2_ climatic effects is arguably the most crucial step towards implementing effective mitigation policies. This primarily includes contrails (the visible trail of ice crystals behind aircraft in cold and humid atmospheric conditions), and NO_x_ (emissions of nitrogen oxides that affect ozone and methane concentrations). A number of experts were invited to share recent findings on our current understanding and uncertainties in this field, in relation to both CO_2_ and non-CO_2_ induced warming.

### Prof. Steven Barrett (Massachusetts Institute of Technology (MIT))

Prof. Barrett pinpointed three primary challenges in mitigating aviation’s climate impact: decarbonisation, reducing contrail warming, and lowering NO_*x*_ emissions. He highlighted the insufficiency of relying solely on aircraft efficiency improvements, succinctly noting, “We can’t efficiency our way to net-zero”. Barrett advocated for SAFs derived from bioenergy crops, underscoring the need for comprehensive life-cycle assessments and cautioning against the biomass production scalability issues, noting, “At some point, there won’t be enough biomass in the world to continue to enable aviation to grow”.

Barrett presented two prospective fuel strategies: hydrocarbon fuels with carbon loop closure and hydrogen as a carbon-free alternative. He stressed the critical role of green electricity and hydrogen production, stating, “There is nothing we can do wrong now by investing in green electricity, biomass to liquid (BtL) and hydrogen.”

In addressing contrail management, Barrett highlighted the uncertainties in measuring their climate impact. He supported the use of satellite-based and deep learning methods^10^ and referred to a study demonstrating significant contrail reduction during the COVID-19 pandemic^11^. He also pointed to MIT’s development of a “real-time contrail avoidance tool”, trialled with Delta Air Lines, as an effective and cost-efficient strategy, concluding, “Contrail avoidance is potentially the cheapest and fastest way to mitigate and roll back the climate impacts of aviation.”

Finally, Barrett suggested novel solutions for NO_*x*_ emission reduction, including electrified aircraft architectures and advanced post-combustion emissions control systems, underscoring their significance in enhancing public health and environmental sustainability in aviation.

### Dr. Roger Teoh (Imperial College London)

Dr. Teoh presented his research on contrail science using the Global Aviation Emissions Inventory based on the ADS-B (GAIA) dataset, analysing contrail properties and their climate forcing from 2019 to 2021^12^. He focused on radiative forcing (RF) as one of the main metrics for climate effects, which is the change in radiative balance at the top of the atmosphere resulting from an ensemble of contrails. His findings indicated a global annual mean net RF from contrail cirrus at 62.1 mW m^−2^, lower than previous estimates^13^. The study highlighted regional RF variations, with Europe, the US, and the North Atlantic having the highest values, while East Asia and China approximated the global mean.

In the open-source repository “pycontrails”^14^, which incorporates the Contrail Cirrus Prediction Model^15^, contrails are defined as persistent if they last beyond the wake-vortex phase. Globally, about 5% of annual flight distances formed persistent contrails in 2019. He noted, “In the subtropics such as East Asia, about 1.7% of the flight distance flown formed persistent contrails.” However, “between 2006 and 2018, most of the global growth in flight distance flown occurred in Asia, e.g. over China and India, where persistent contrails are less likely to form and have a shorter lifetime”. Previous scientific consensus assumed that the increase in global annual mean contrail net RF is proportional to the global increase in annual flight distances^12^ (Fig. 2).

Overall, he found that 24% of all flights formed persistent contrails. Just 2.7% of all flights account for 80% of the global annual energy forcing, further quantifying the cumulative energy trapped in the atmosphere by contrails^12^. Teoh stressed, “On average, if you form a persistent contrail, there is a 70% probability that it is more likely to be warming and the average warming effect is an order of magnitude larger than flights that formed cooling contrails.” His findings are now being tested operationally, in pilot programmes aimed at steering airlines away from ice-supersaturated regions (ISSRs), to avoid persistent contrail formation.

### Dr. Alexandru Rap (University of Leeds)

Dr. Rap gave a talk on the global contrail and NO_*x*_ climate impact from current and alternative fuel aircraft, SAFs, liquid hydrogen (LH_2_) and hydrogen fuel cells (FCs). Initially, a methodology was presented to predict the thermodynamic conditions required for contrail formation and persistence, for kerosene, SAFs, LH_2_ and FCs^16^. It is found from the analysis that LH_2_ combustion and FCs will produce fewer but larger ice crystals compared to kerosene. LH_2_ combustion produces  ~2.6 times as much water vapour emissions compared to kerosene, however there are no particulates formed. Therefore, ice crystal concentrations will largely be defined by background aerosol particle concentrations.

Rap found substantial increases in global contrail cover from LH_2_ and FCs compared to kerosene and SAFs. This is due to the increased water vapour emissions, and hydrogen contrails forming at higher temperatures (lower altitudes). It is predicted, however, that hydrogen contrails induce less warming due to different ice crystal properties. Significantly, global contrail cirrus warming was found to be highest for kerosene, followed by SAFs, FCs and LH_2_. Analysis of global NO_*x*_ climate impact showed an approximately linear response of NO_*x*_ climate impact reduction with reduced NO_*x*_ emissions.

### Dr. Irene Dedoussi (Delft University of Technology)

Dr. Dedoussi presented her latest research on sustainable aviation decision-making in an evolving atmosphere. Firstly, Dedoussi addressed non-linear atmospheric modelling of aviation air quality and climate impact, through two studies using different modelling techniques: Finite difference (suitable for estimating source-oriented sensitivities) and Lagrangian (can track emissions through simulation of individual air parcels).

In the finite difference study, aircraft emissions were perturbed in three continental regions, Europe, Asia, and North America. An important finding from this study was that efforts to mitigate landing and take-off emissions will have twice the effects over Europe than they will over North America^17^.

In the Lagrangian study, air parcels were tracked to see how aviation NO_*x*_ cruise emissions lead to ozone formation and the associated radiative forcing. It was found that the resulting RF is also highly dependent on season and region of emission^18^.

The second half of the talk was on estimating aviation emissions using real-world aircraft activity data and performance and emissions modelling^19^. Dedoussi’s analysis highlighted the increasing NO_*x*_ emissions index over time and showed how emissions can vary significantly, depending on aircraft engine assignment. The talk concluded by highlighting that the evolving aviation emissions and strategies targeting their reduction should be addressed in the context of this spatially varying atmospheric response.

## Optimising aircraft operations

Regulatory bottlenecks result in the slow adoption of new technologies and alternative fuels in aviation, compared to other sectors. Therefore, it is imperative that the industry seeks out the most efficient and climate-optimal ways to operate the existing global fleet. This issue was explored at ACIM2030, from a scientific, operational and commercial perspective.

### Dr. Adam Durant (SATAVIA)

Dr. Durant highlighted the significant climate impact of aviation contrails, noting that they nearly double the impact of direct CO_2_ emissions, amounting to an annual impact of 652 MTCO_2_e (GWP_100_)^13^. SATAVIA’s approach to contrail management includes improved prediction of ISSRs and pre-tactical flight plan adjustments (Fig. 3).

The company is conducting trials with airlines to address the substantial impact of contrail clouds. Durant discussed efforts to transform post-flight verified climate benefits into tradable carbon credits^20^. The EU ETS is planning to measure aviation’s non-CO_2_ impact from 2025; financial penalties may come in some years after that time.

Their technology, a digital twin of the atmosphere optimised for cruise altitude weather, has shown high reliability in predicting ISSRs (manuscript in preparation). These predictions are compared to in-situ measurements within the IAGOS project^21^. A case study with Etihad demonstrated the real-world effectiveness of this approach^22^.

### Kieran Tait (University of Bristol)

Tait gave a presentation on the potential to mitigate non-CO_2_ climate impact through formation flight and controlled aircraft exhaust plume overlap. Aircraft emission species are entrained within the vortical structure of the wake for 2–12 h post combustion. Thus, species concentrations are elevated locally, until the plume disperses due to wind shear and advection effects. This temporary accumulation of emissions is responsible for initiating contrail formation from excess particulates and also causes an initial drop in ozone production due to high concentrations of NO_*x*_^23^.

In busy airspace, when multiple exhaust plumes overlap, such effects are exacerbated. The formation of two or more overlapping contrails leads to mutual inhibition of their growth and persistence due to the uptake of finite ambient water vapour. Furthermore, when NO_*x*_ concentrations are consistently elevated, this can lead to a NO_x_-saturated regime, in which additional emitted NO_*x*_ becomes less efficient at producing ozone, thus limiting its warming potential. These two effects can be exploited in a controlled plume overlap scenario.

Tait is currently collaborating with Breakthrough Energy to integrate a version of the STOCHEM-CRI chemistry transport model^24^ into pycontrails, and plume overlap effects will be explored in an upcoming 2024 study.

## Alternative fuels and technology developments

The aviation industry has achieved continual efficiency improvements through changes to aircraft/engine design and new technologies. In recent years, however, progress on efficiency and fuel burn reduction has stalled, while growth continues unabated. Two speakers discuss the importance of continuing to push the boundaries in this area, whilst also advocating for the switch from conventional jet fuel to SAFs in the short term.

### Henry Edwards (Airbus)

Edwards gave a talk on supporting Airbus efforts to maximise aviation climate impact reduction this decade, through innovation in fuel systems and collaboration with the research community. Edwards highlighted some key challenges manufacturers face in the SAF roll-out: adverse behaviour of elastomers used in fuel seals under a change in aromatic content, equipment clearance and material compatibility throughout the supply chain, fuel energy content affecting aircraft performance and lastly, the debate over 100% drop-in vs. non-drop-in SAF. The most significant challenge presented, however, is the availability of truly sustainable feedstocks^4^.

To conclude the talk, Edwards put forward a number of thought-provoking concepts around innovative ways for the industry to reduce emissions in the short term. This included catalytic reduction of NO_*x*_ through improvements to engine design, the use of fuel additives to inhibit corrosion, enhance lubricity, dissipate static, and the prospect of a “well-to-wake-to-climate” model to conduct detailed lifecycle analyses on aviation’s environmental impact.

### Prof. Jonathan Cooper (University of Bristol)

Prof. Cooper presented the potential to reduce aviation climate impact this decade through innovative aircraft technologies. This approach was discussed in the context of optimising the Breguet Range equation^25^, such as increasing the lift-to-drag ratio (aerodynamic performance), reducing structural weight, improving engine efficiency and flying higher and faster. Aerodynamic advancements could include novel design configurations such as high aspect-ratio wings with structural weight minimised through active flutter suppression and improved load alleviation through folding/morphing wingtips^26^ and the use of aeroelastic tailoring designs. Technology such as electric taxiing could serve as intermediate solutions this decade (see the section “Outlook”).

## Policy developments

It is evident that with the inclusion of non-CO_2_ effects, aviation climate impact is a complex, nonlinear problem that requires a multifaceted mitigation approach. As such, rigorous policy-making and market incentivisation are required, to ensure rapid roll-out of effective measures as soon as they become scientifically and technologically available.

### Dr. Bethan Owen (Manchester Metropolitan University)

Dr. Owen’s presentation highlighted the International Civil Aviation Organization’s (ICAO’s) crucial role in global collaboration and explained the role of the “Emissions Technical Group”, which she co-chairs, responsible for setting emissions and regulatory standards. She mentioned both CO_2_ and non-CO_2_ emissions; however, she concentrated on the importance of CO_2_ due to its long atmospheric life, “what we do now will remain in the atmosphere for many years”. She added that “CO_2_ equivalencies [...] can be very misleading depending on the time horizon we are looking at”.

At ICAO, operational contrail avoidance falls under the responsibility of another working group, i.e. the “Airport and Operations Group”. Dr. Owen expressed concerns about the readiness for implementing operational strategies in this area, as “there are some fundamental things that we need to improve upon before operationally going for it.”

The 2025 Committee on Aviation Environmental Protection (CAEP) meeting, she noted, will be crucial for tightening CO_2_ standards for new aircraft from 2029/2030, balancing emissions reductions with practical and technological feasibility, and considering noise and CO_2_ emission trade-offs.

### Prof. Ian Poll (Cranfield University)

Prof. Poll discussed the significant role of aviation in both contributing to and solving environmental issues, especially regarding contrail management. He highlighted aviation’s dual role, emphasising its vital contribution to global GDP and particularly its importance in developing economies. Poll stated, “In terms of the environment, aviation is both part of the problem and part of the solution.” and “The developing economies and the poorest countries have the most to gain from aviation.”

Poll focused on the impact of contrails, especially persistent ones, in contributing to climate change, noting that their effect is more significant than CO_2_ emissions in the aviation sector. He advocated for contrail management as an immediate and feasible solution, stating, “The real objective is contrail management—not contrail avoidance. Avoiding the warmers and generating more coolers is the real target.” This approach, he mentioned, doesn’t require new technology or substantial investment and is within the airlines’ control.

## Outlook

The short-term focus of the ACIM2030 conference meant that mitigation of non-CO_2_ climate impact through route optimisation and alternative fuel usage dominated the day’s discussions. Such measures are widely viewed by key industry players as “the elephant in the room” or “low-hanging fruit”, and thus can be acted on with the utmost immediacy. However, great uncertainty in the science still remains, with short-term intervention on contrail avoidance and targeted SAF usage being part of a hotly contested debate, amongst the aviation climate science community^27^.

What is the acceptable level of scientific uncertainty for action to be taken? What will it take to reach an agreement on a suitable metric for the comparison of aviation climate forcers? Who will be the arbiter on such matters? To bring about the drastic action necessary, these are topics we must find a consensus on.

Dr. Rap believes that, before we switch to alternative fuels, we must first increase our understanding of aerosol–cloud interactions, improve the representation of ISSRs and investigate the sensitivity of climate-optimal routing to existing uncertainties and local and regional-scale weather variations.

Prof. Cooper highlighted the possible emissions reduction that could be achieved through mitigating aircraft taxiing emissions. Between 2% and 14% of fuel burn happens during the taxiing stage^28^, meaning fully electric taxiing and changes to ground operations should be a focal point for action this decade. Furthermore, retrofitting components onto existing aircraft, such as winglets and folding wing tips, could help bring about increases in efficiency over much shorter timescales than the lifetime of today’s fleet.

The main political challenge, according to Prof. Poll, lies in the lack of financial incentives for airlines to implement contrail management strategies. He warned of the risks of inaction; in the event of an ecological disaster, the lack of a prompt response from the aviation sector could lead to government-imposed demand management, causing significant damage to the industry and its dependent sectors. Poll urged the aviation industry to proactively adopt contrail management strategies to mitigate these risks.

If we are to see drastic climate impact mitigation this decade, it is crucial that the research community: prioritises collaboration with industry and other academic/research institutions worldwide, focuses on aforementioned open issues to reduce uncertainties, clearly formulates an acceptable level of certainty for large-scale intervention, develops roadmaps to clearly specify aviation climate impact mitigation trajectory (including non-CO_2_ effects).
